# The role of glutamate dehydrogenase in the ageing brain

**DOI:** 10.3389/fphar.2025.1586655

**Published:** 2025-04-28

**Authors:** Tao Zhou, Haichuan Wang

**Affiliations:** ^1^ Department of Pharmaceutical and Medical equipment, Bayi Orthopedic Hospital, China RongTong Medical Healthcare Group Co. Ltd., Chengdu, China; ^2^ Department of Paediatrics, Sichuan Academy of Medical Science & Sichuan Provincial People’s Hospital, School of Medicine, University of Electronic Science and Technology of China, Chengdu, China

**Keywords:** glutamate dehydrogenase, ageing brain, Parkinson’s disease, Alzheimer’s disease, glutamate metabolism

## Abstract

The homeostasis of glutamate, the primary excitatory neurotransmitter in the brain and is crucial for normal brain function. The mitochondrial enzyme glutamate dehydrogenase (GDH) connects the multifunctional amino acid glutamate, which is intimately related to glutamate metabolism, to the Krebs cycle. As a result, GDH reglutes the synthesis and uptake of the chemical messenger glutamate in neuroendocrine cells, playing a crucial role in the metabolism of proteins and carbohydrates. Nonetheless, brain ageing and numerous neurodegenerative diseases, including Parkinson’s disease and Alzheimer’s disease, have been linked to GDH malfunction or dysregulation. In this review, we summarize the dynamics of GDH levels in the ageing brain and provide additional details about the role of GDH in the ageing brain. Understanding the metabolic mechanisms underlying glutamate homeostasis in the aging brain and how GDH regulates glutamate-dependent metabolic processes at synapses may lead to novel therapeutic approaches for neurodegenerative and psychiatric disorders, potentially slowing the aging process and promoting brain regeneration.

## Introduction

Glutamate dehydrogenase (GDH) is expressed mostly in the brain, liver, kidney, and pancreas and is located in the mitochondrial matrix of humans. As a hexameric structure, functional GDH consists of two sets of trimers, each containing approximately 500 amino acids. Each monomer has a binding site for guanosine triphosphate (GTP) and another for nicotinamide adenine diphosphate hydride (NADH)/adenosine diphosphate (ADP); this structure allows six GTP molecules, six ADP molecules, and six NADH molecules to bind to the GDH hexamer at its allosteric binding sites. When a substrate interacts with two GDH trimers, a projecting domain that resembles an antenna connects the subunits (Nassar et al., 2019).

Numerous small molecules allosterically regulate GDH in animals. GTP and ATP are the primary allosteric inhibitors. GTP binds to the allosteric domain above the catalytic domain at the bottom of the antenna to block GDH catalytic activity and stop the release of glutamate oxidative products, which is a rate-limiting step. By inhibiting the release of NAD(P)H, an oxidative product of glutamate, NADH can bind to the NADH/ADP binding site and strengthen the inhibitory effect of GTP on GDH activity. Furthermore, diethylstilbestrol (DES), palmitoyl-CoA, and steroid hormones are also inhibitors of mammalian GDH, although the sites where they bind are unclear. Finally, green tea contains a polyphenol called epigallocatechin gallate (EGCG), which likewise functions as an allosteric inhibitor by competing with ADP (Li et al., 2014). In contrast, leucine and ADP are the main allosteric activators of GDH. In contrast to GTP, ADP activates GDH. By binding to the NADH/ADP binding site and preventing GTP binding, ADP can activate GDH. Leucine is both an allosteric activator and a catalytic substrate of GDH; although it has a distinct binding site, its activation mechanism is comparable to that of ADP (Li et al., 2014).

## GDH expression and function

**FIGURE 1:**
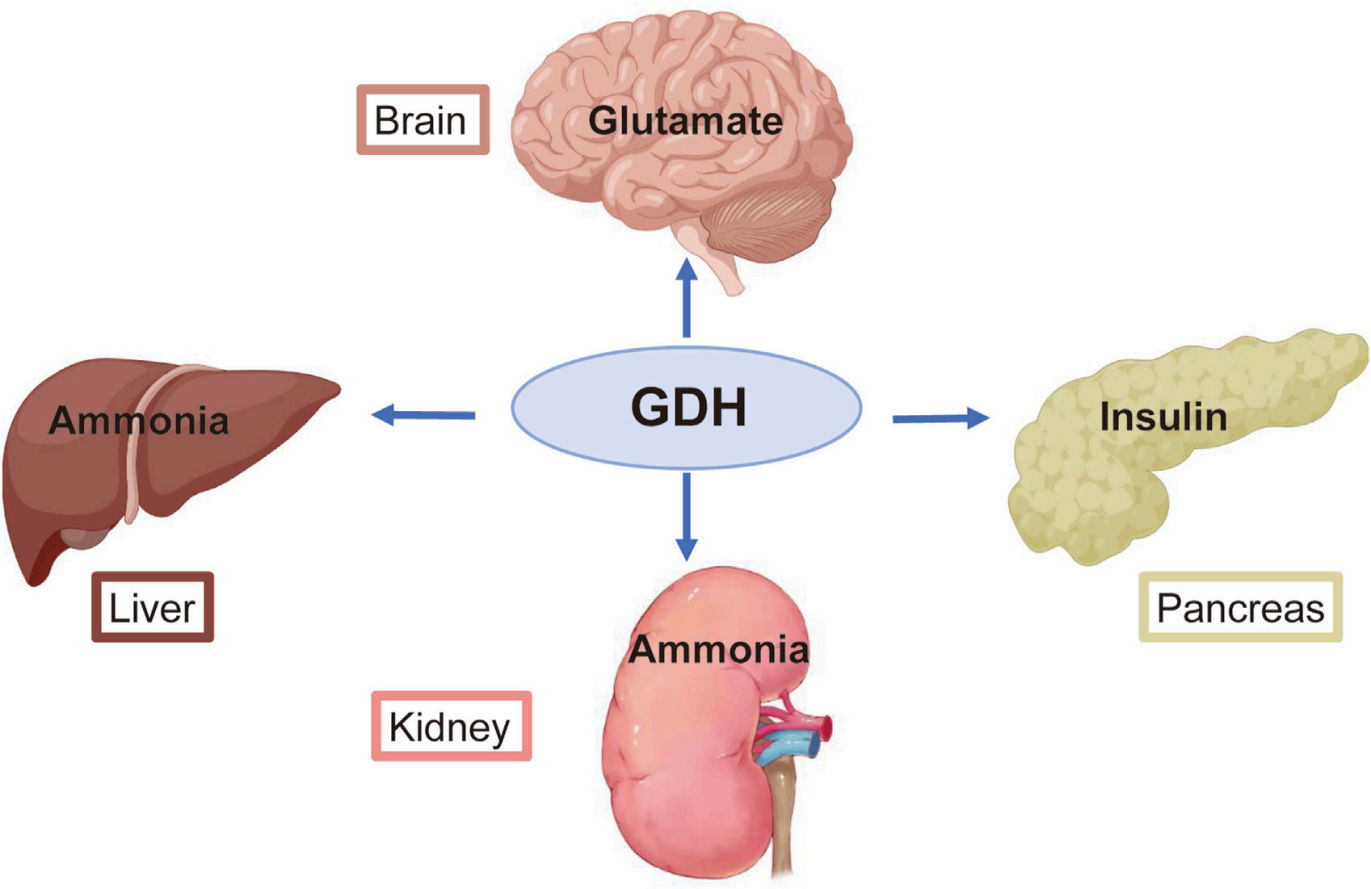
Primary roles of glutamate dehydrogenase (GDH) in several organs. GDH helps the brain recycle glutamate and regulates the production of the primary excitatory neurotransmitter. GDH is essential for the metabolism of ammonia in the liver and kidney. GDH activity affects the rate of insulin release from pancreatic β-cells.

GDH activity varies greatly in various mammalian tissues (Botman et al., 2014; Treberg et al., 2014). High enzyme levels are present in the liver, brain, kidney, pancreas, adrenal glands, and placenta (Rothe and Storm-Mathisen, 1995; Spanaki et al., 2014) (Figure 1). The liver, where the enzyme comprises approximately 1% of the total protein, has the highest GDH-specific activity; GDH activity is lower in other mammalian organs (Botman et al., 2014; Treberg et al., 2014). According to estimates, the GDH level in the human kidney and brain is between 20% and 25% of that in the liver (Spanaki et al., 2010). Recently, studies have employed IHC to examine human tissues using antibodies that detect both human GDH isoenzymes. They discovered that all of the hepatocytes in the human liver express GDH quite abundantly. All cells of the pancreatic parenchyma in humans, including acinar cells and the endocrine cells of Langerhans islets, express GDH (Spanaki et al., 2014). In addition, the epithelial cells of the proximal convoluted tubules are where GDH is mostly localized in the human renal cortex (Spanaki et al., 2014; Spanaki et al., 2012). Finally, GDH is present in both Sertoli and Leydig cells in the human testis (Spanaki et al., 2014; Spanaki et al., 2010). However, spermatogonia, spermatocytes, and spermatozoa, i.e., germ cells, are negative for GDH expression (Spanaki et al., 2014; Spanaki et al., 2010).

**FIGURE 2:**
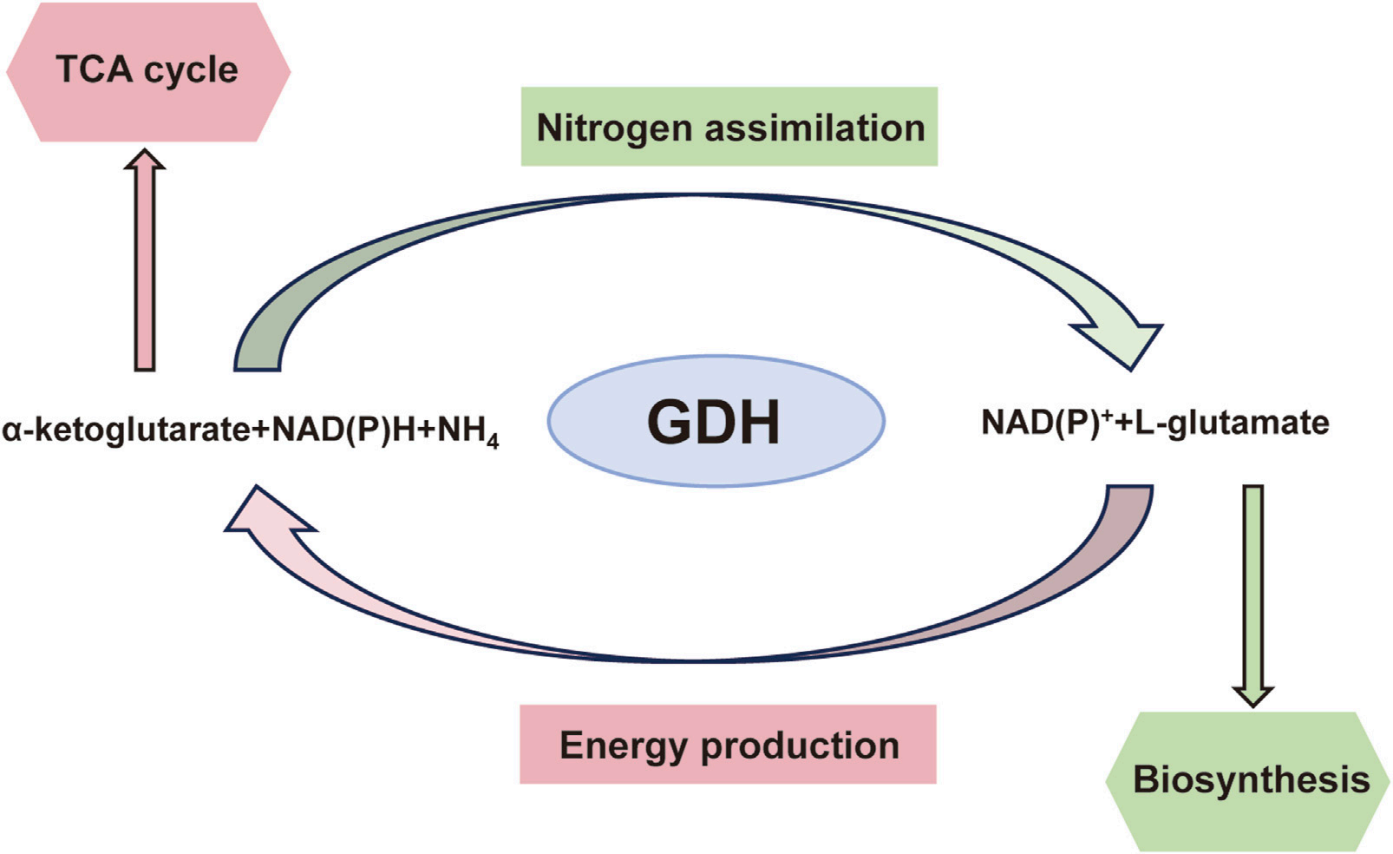
Diagrammatic representation of glutamate dehydrogenase-catalysed processes and their overall metabolic importance.

Under normal circumstances, GDH is a major regulator of the metabolism of amino acids and ammonia in the human pancreas, liver, and brain. GDH uses NAD(P)^+^ as a coenzyme to catalyze the oxidative deamination of glutamate to produce alpha-ketoglutaric acid (α-KG) and ammonia via the following reversible reactions: glutamate + NAD(P)^+^ ↔ α-KG + NH_3_ + NAD(P)H. α-KG is used in the tricarboxylic acid (TCA) cycle to produce adenosine triphosphate (ATP) (Figure 2). In islet β cells, the brain, and renal tubular cells, the reaction is directed primarily towards the oxidative deamination of glutamate because these cells and tissues contain high glutamate and low α-KG/NH3 levels (Komlos et al., 2013).

GDH aids in preserving the NH4^+^ concentration in the kidneys and several other organs (Cooper and Jeitner, 2016; Treberg et al., 2010). Additionally, the enzyme plays a role in the mechanism by which pancreatic β-cells secrete insulin (Sener et al., 1980). GLUD1 mutations reduce the sensitivity of the enzyme to its inhibitor GTP, which fuels hyperinsulinaemia/hyperammonaemia (HI/HA) syndrome, a condition characterized by elevated blood NH4^+^ levels and low glucose levels (De Lonlay et al., 2001; MacMullen et al., 2001). Pancreatic β-cells expressing mutant GDH produce 2-oxoglutarate in a dysregulated manner, which is linked to the release of insulin (Fahien and Macdonald, 2011).

Mutations in short-chain 3-hydroxyacyl-CoA dehydrogenase (SCHAD), which is involved in the oxidation of fatty acids, can also result in hyperinsulaemia and hypoglycaemia (Kapoor et al., 2009; Molven et al., 2004). Recently, GDH from pancreatic islets was shown to be bound by SCHAD, which inhibits its activity (Li et al., 2010). However, mutated SCHAD is incapable of binding to GDH. The subsequent activation of pancreatic GDH causes incorrect insulin release (Li et al., 2010), comparable to that observed with GLUD1 mutations, which impair GDH inhibition by GTP (Nissim, 1999; Treberg et al., 2010).

Research on ADP-ribosylation suggested that this alteration also plays a part in mediating the effect of GDH on insulin synthesis. For example, GDH in pancreatic β-cells is rendered inactive by SIRT4-dependent ADP-ribosylation, which restricts the release of insulin (Haigis et al., 2006). Under low-glucose conditions, the inactivation of GDH may be reversed, leading to an increase in blood insulin levels and pancreatic GDH activity. Under calorie restriction, reduced ADP-ribosylation of GDH was observed, which was linked to increased insulin levels and increased GDH activity (Guan and Xiong, 2011).

## Genetics of GDH

The well-preserved 45-kb gene GLUD1, which is divided into 13 exons, encodes GDH (Michaelidis et al., 1993). While GLUD1, which is found only in hominoids, is expressed in multiple tissues (Plaitakis et al., 1980), its isoform, GLUD2, is expressed exclusively in the brain and testicular tissues (Shashidharan et al., 1994). The sole isoform in rodents that encodes GDH is GLUD1 (Burki and Kaessmann, 2004). Recent studies have revealed that astrocytes and testicular supporting cells exhibit significant levels of GLUD2 expression (Spanaki et al., 2010). GLUD2 is an intronless X-linked gene (Shashidharan et al., 1994) that evolved fewer than 23 million years ago via the retrotransposition of a spliced mRNA from the GLUD1 gene, which has an intron, in hominoid progenitors. The alterations in amino acids that confer the distinct brain-specific characteristics of the GLUD2-derived enzyme occurred when the size of the brains of ancestors of humans and great apes increased. By permitting greater neurotransmitter transit and clearance, GLUD2 may have contributed to improved brain function in both humans and apes. Therefore, GLUD2 may have played a role in hominoid evolution and the development of an increased cognitive capacity (Burki and Kaessmann, 2004).

The pH dependence of human GDH isoenzymes (hGDH1 and hGDH2), which are encoded by distinct genes (GLUD1 and GLUD2), varies. In the 2-oxoglutarate amination process, hGDH2 shifts the pH optimum from 8.0 to 7.5, in contrast to hGDH1 (Kanavouras et al., 2007; Plaitakis et al., 2003). This distinction is thought to be significant in astrocytes, whose absorption of synaptic glutamate linked to OH ion counter-transport acidifies their cytoplasm and mitochondrial matrix (Poitry et al., 2000).

ADP activates both isoenzymes; however, ADP activation of hGDH2 is more noticeable. The two enzymes also differ in their nucleotide-dependent regulation (Plaitakis et al., 2000). The amplitude of the effect at saturating ADP concentrations (approximately 1 mM) is ten times greater for hGDH2 than for hGDH1; specifically, the SC50 value for ADP, the ADP concentration eliciting 50% of the maximum activation, is roughly three times higher for hGDH2 (58.7 µM) than for hGDH1 (17.0 µM) (Kanavouras et al., 2007). Similarly, the impact of leucine, another well-known allosteric activator, is approximately ten times greater for hGDH2 than for hGDH1, even though the two hGDH isoenzymes' SC50 values and their decreased activity with the addition of ADP are comparable (Kanavouras et al., 2007).

Cho et al. reported the kinetics of the two different GDH forms from the bovine brain (bGDH1 and bGDH2) (Cho et al., 1995). The authors use the terms isoform and isoprotein as synonyms for bGDH1 and bGDH2. We refer to these GDHs as the enzyme forms since the structural information to categorize them as isoforms or isoenzymes using the previously stated categories is currently insufficient. bGDH1 and bGDH2 were separated from the whole homogenate (Cho et al., 1995). The kinetic characteristics of bGDH1 and bGDH2 were measured at a fixed ADP concentration (1 mM) and are similar to those of the hGDH1 and hGDH2 isoenzymes at intermediate ADP saturation (0.1–0.25 mM). Specifically, both hGDH1 and bGDH1 have a higher Km for glutamate and a lower Km for 2-oxoglutarate than do hGDH2 and bGDH2. The two human and bovine isoenzymes also exhibit the same differences in the Km for ammonium, and under similar conditions, allosteric activation by ADP was more evident for bGDH2 than for hGDH2 (Cho et al., 1995). In addition, the high sensitivity of the hGDH2 isoenzyme to dilution, as previously mentioned, is also reflected in the higher Vmax for bGDH1 than for bGDH2 (202 and 124 μmol/min per mg of protein, respectively, in the presence of 1 mM ADP). Overall, kinetic studies indicate that the two enzyme forms of GDH identified in the bovine brain are functionally similar to the two human GDH isoenzymes.

## GDH in brain development

Since glutamate is the primary excitatory neurotransmitter in the central nervous system (CNS), mitochondrial function and glutamate metabolism are closely related in the brain (Baudry and Lynch, 1979). Specifically, mitochondria control the energy state of the brain, the use of metabolic substrates, and the detoxification of excess neurotransmitters.

Target neurons are depolarized by glutamate produced by active synapses via certain receptors (Mayer et al., 1984; Spreafico et al., 1994). Glutamate is a quick-acting neurotransmitter, but it also has long-lasting effects on the structure and function of neurons as a signalling molecule. Glutamate neurotransmission plays a significant role in modulating synaptic activity during nervous system development (Cohen and Greenberg, 2008; Ghiani et al., 2007), as well as in learning and memory construction and the gathering and storing of new knowledge (Cotman et al., 1988; McEntee and Crook, 1993).

Enzyme histochemistry has shown that GDH is present in rat hippocampal dendritic layers and that GDH activity increases in tandem with the postnatal maturation of synaptic structures (Schünzel and Wolf, 1982). Similar increases in neuronal GDH staining were observed in subsequent studies of the rat hippocampal region during postnatal development, namely, in the stratum lacunosum-moleculare and the molecular layer of the dentate gyrus in the hippocampus (Kugler and Schleyer, 2004; Rothe and Schünzel, 1990). Significant increases in GDH levels have also been observed in the rat cerebellum in the late postnatal stage, an increase that is believed to be a reflection of synaptogenesis and neuronal development (Wolf and Schünzel, 1987; Wolf et al., 1986). In particular, GDH activity increases in the cerebellar cortex in the molecular layer, granule cell bodies, internal granule cell layer, cerebellar glomeruli, and Purkinje cell perikarya to a lesser extent. Studies that have measured GDH activity and exogenous glutamate use in primary neuronal cultures from the rat ventral mesencephalon have shown that GDH activity is approximately four times higher in mature (12-day-old) cultures than in immature (4-day-old) cultures and have identified a significant correlation between exogenous glutamate use (added to the medium at 1.2 mM) and GDH activity in cultured cells (Plaitakis and Shashidharan, 2000). Furthermore, compared with mature cultures, immature cultures secrete significantly more alanine and aspartate into the medium, indicating that the transamination route is the primary mechanism by which exogenous glutamate is metabolized in these cells. Together with the previously mentioned results showing significant increases in GDH activity in the growing brain, these observations imply that the growth of nerve terminals and synaptic connections coincides with significant increases in glutamate use (away from transamination).

## GDH in the ageing brain

Developmental synaptic pruning, which is essential for honing neurons, progressively results in cumulative, nonpathological synaptic degradation over time, which has an irreversible effect on all maturing organisms. The capacity of an individual to successfully adjust to their surroundings is inevitably hampered by this natural ageing process (Diniz and Crestani, 2023). Ageing is a major risk factor for the development of mental and neurological diseases (Cole et al., 2019; Hou et al., 2019). Changes in neuroplasticity or cellular changes that directly affect plasticity pathways may be partially responsible for the loss of cognitive performance associated with ageing. Numerous studies have shown that as people age, the glutamatergic system changes, which can result in synaptic dysfunction, neuronal damage, or even death (Cox et al., 2022; Segovia et al., 2001). Chronic inflammation plays a major role in neurodegenerative disorders and brain ageing (Godbout and Johnson, 2009; Hirsch and Hunot, 2009). Accordingly, research has shown that as mice age, both wild-type (Wt) and transgenic (Tg) mice with CNS-Glud1 overexpression may exhibit elevated expression of genes linked to neuroinflammation (Wang et al., 2014). Another study revealed that only Tg model mice exhibited a chronic inflammatory response when the hippocampal regions of 9-month-old CNS-Glud1 Tg and WT mice were examined (Wang et al., 2010). These findings could suggest a possible connection between neuroinflammation in the ageing brain and variations in GDH expression.

As mentioned previously, GDH activity fluctuates throughout life and differs across various brain regions. Modest GDH activity is detected in the central and peripheral nervous systems of rats at birth. Nonetheless, GDH activity increases significantly during the postnatal developmental stage, with earlier increases in the medulla and a rough doubling in the cerebellar cortex and fivefold increase in the hippocampus (Rothe et al., 1983). The development of brain regions with high glutamatergic activity results in elevated GDH activity (Rothe et al., 1983). On the other hand, a different study revealed that as male albino rats aged, the tissues throughout the brain presented a decrease in GDH activity (Rajeswari and Radha, 1984). GDH reactivity in the hippocampal regions of juvenile, adult, and elderly rats has also been assessed. In young rats, the stratum oriens of the CA1–CA4 regions in the hippocampus has the highest enzymatic reactivity. The molecular layer of the fascia dentata and the layer of mossy fibres are next in descending order. In practically every stratum analysed, adult rats exhibit stronger enzymatic reactivity than do juvenile rats. Except for the stratum oriens of the CA1–CA4 fields, where enzymatic reactivity is higher in older rats than in adult rats, GDH reactivity is lower in older rats than in adult rats (Bronzetti et al., 1990). Similarly, previous research has shown that in rats, GDH activity is detected earlier in the medulla oblongata and pons than in other brain regions (Leong and Clark, 1984). In adults, these areas present the greatest activity, with the midbrain and hypothalamus being the second and third most affected areas, respectively.

Studies in humans and rodents have consistently shown a loss of synaptic connections and neurons along glutamatergic neurotransmission pathways with aging (Francis, 2003; Morrison and Hof, 2007). These losses mostly impact areas of the brain, such as the cerebral cortex and hippocampus, which are also affected in Tg mice that overexpress Glud1. Because glutamate transport into glial cells or neurons is reduced in the brains of old mice, the loss of synapses and neurons in these areas results in increased amounts of extracellular glutamate (Nickell et al., 2005; Zoia et al., 2004). Tg mice overexpressing Glud1 have been used to investigate the connection between the ageing process and glutamate-induced metabolic responses (Choi et al., 2014). The findings of that study revealed that the hippocampus and striatum of Tg mice differed from those of WT mice at different ages in terms of the amount of neurochemicals, including lactate (Choi et al., 2014).

Neurons appear to be specifically susceptible to glutamate release and overproduction, as well as ageing. Wang et al. reported that Glud1 Tg and WT mice have different gene expression patterns (Wang et al., 2014); additionally, regardless of whether a mouse was Tg or WT, dynamic changes in the expression of certain genes in the hippocampus were observed during ageing through comparisons across several age periods spanning from 10 days to 20 months of life. Overall, the hippocampal transcriptome was more significantly affected by Glud1 overexpression in mature and aged mice than in young mice. Importantly, the Gene Ontology (GO) categories of genes with altered expression in Tg mice overlapped with a significant number of GO categories of genes with altered expression in WT mice during ageing, even though the changes in gene expression in the hippocampus of Tg and WT mice did not always follow the same pattern. The expression of genes linked to growth, neurogenesis, process elongation, and neuronal migration decreased in both Tg and WT animals during the developmental, maturation, and ageing phases; additionally, genes involved in neuroinflammation, voltage-gated channel function, and synaptic transmission modulation were activated. In a different investigation, Michaelis et al. observed that in the CA3 area of the hippocampus, Glud1 overexpression does not result in any age-related changes in dendritic structure or neuronal population numbers (Michaelis et al., 2011). In another study, which contrasted anterograde axoplasmic transport in WT mice with that in Tg mice overexpressing the Glud1 gene in CNS neurons, the authors reported that Tg animals presented increased rates of anterograde axoplasmic transport in olfactory neurons *in vivo* and in hippocampal neurons in brain slices *ex vivo* (Lee et al., 2024). Given the links between neurodegeneration and disrupted axonal transport (Berth and Lloyd, 2023) and the ageing process (Milde et al., 2015), additional research is urgently needed.

Notably, GDH plays an epigenetic role in neuronal plasticity. Research has indicated that the α-KG-consuming protein Tet3 (the primary α-KG-dependent dioxygenase in neurons that converts 5-methyl-dC into 5-hydroxymethyl-dC and then into 5-formyl- and 5-carboxy-dC) reroutes mitochondrial GDH, which converts glutamate into α-KG in an NAD + -dependent manner, to the nucleus, indicating the onsite production of α-KG. Additionally, glutamate dehydrogenase has a stimulatory effect on Tet3 demethylation activity in neurons, and neuronal activation increases the level of α-KG. Overall, the GDH–Tet3 interaction may play a role in epigenetic changes during neural plasticity (Traube et al., 2021).

**FIGURE 3:**
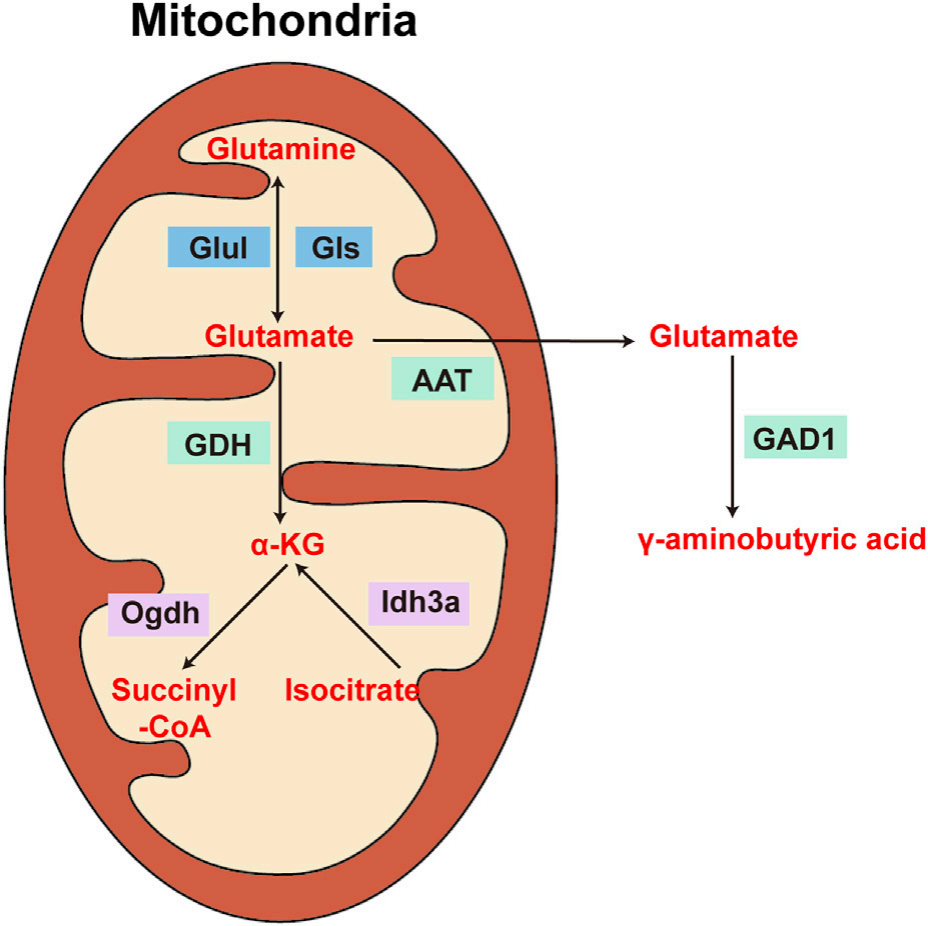
Diagram of the enzymes involved in the metabolism of glutamate, glutamine, α-ketoglutarate, and γ-aminobutyric acid in the brain. Glul: glutaminase; Gls: glutamine synthetase; GDH: glutamate dehydrogenase; Ogdh: α-ketoglutarate dehydrogenase; Idh3a: isocitrate dehydrogenase subunit three alpha; GAD1: glutamate decarboxylase 1.

The cellular dysfunction observed during ageing has been partially attributed to impaired mitochondrial activity (Javadpour et al., 2024; Miwa et al., 2022). Studies have shown that GDH activity can differ among various populations of brain mitochondria, in addition to differences in GDH levels and activity across different brain areas. Subcellular fractionation results revealed that the nonsynaptic mitochondria of the medulla oblongata have approximately double the GDH-specific activity of those of the striatum and cortex; in contrast, the GDH activity in synaptic mitochondria is essentially consistent throughout various rat brain areas (Leong and Clark, 1984). Glutamate metabolism has been assessed in a variety of mitochondrial populations in the brain at different ages during the ischaemia and postischemic healing stages (Ferrari et al., 2018); different cerebral mitochondria exhibit varying levels of GDH activity, indicating different responses to ischaemia, postischemic recovery, and ageing. The ability of glutamate to increase synaptic adaptability during the recirculation phase of brain ischaemia may account for the diverse impacts on brain mitochondria. Therefore, depending on where they are located in the presynaptic and postsynaptic compartments, separate nonsynaptic and intrasynaptic mitochondria have variable metabolic properties that result from differing energy needs. However, in another study, GDH activity was shown to decrease with age in both synaptic and nonsynaptic mitochondrial populations in the brains of rats (Deshmukh and Patel, 1980) (Figure 3).

**FIGURE 4:**
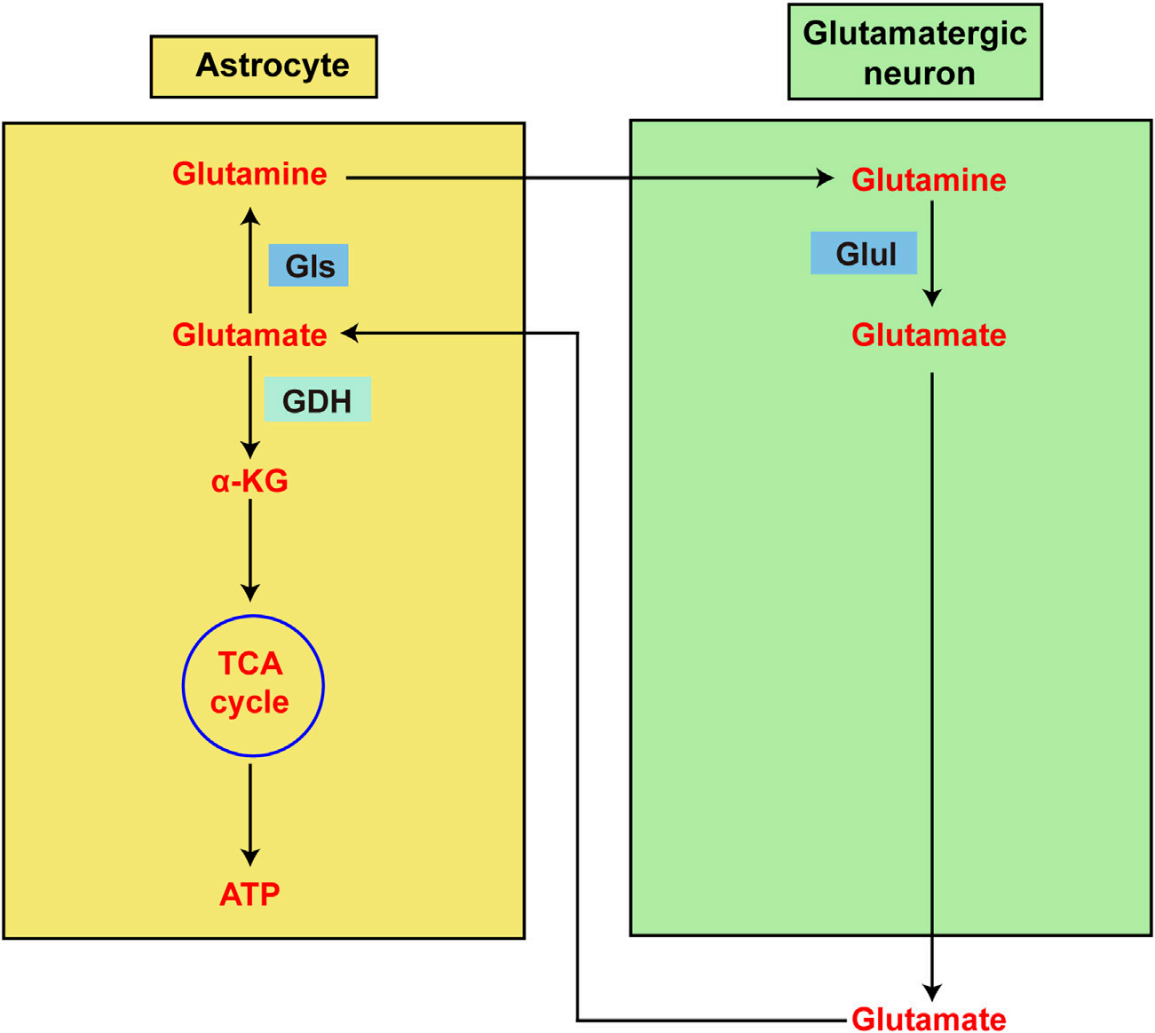
Diagram showing the metabolism of glutamate in astrocytes and glutamatergic neurons. Glutamate is the primary excitatory neurotransmitter in the central nervous system (CNS). However, glutamate is also intimately linked to energy metabolism in the brain. Astrocytes are primarily responsible for intersynaptic glutamate elimination following glutamatergic transmission. Glutamate can be recycled back to neurons after being aminated to glutamine by astrocytes. Alternatively, glutamate dehydrogenase (GDH) may deaminate glutamate to α-ketoglutarate prior to continued oxidation in the TCA cycle, which promotes ATP production.

Changes in GDH levels have been observed in brain cells and other cell types with ageing, findings that may be associated with changes in CNS metabolism. According to previous studies, leukocyte GDH activity is lower in older people than in younger people, suggesting that GDH activity may play a role in ageing (Kravos and Malesic, 2010). However, another study revealed that the overall activity of GDH in lymphocytes increases with age, which may represent an adaptive reaction related to glutamatergic neuron loss (Iwatsuji et al., 1989). The regulatory mechanism of GDH is most likely linked to the regulation of glutamine metabolism (Iwatsuji et al., 1989). However, numerous animal studies have shown that GDH activity in the brain increases with age and decreases with senescence (Mahapatro and Patnaik, 1993). However, GDH activity in the rat liver does not vary with age (Prabhakaram and Singh, 1988). These findings suggest that different cell types exhibit different changes in GDH activity as they age (Figure 4).

Mammalian GDH is modulated by high levels of progesterone and oestrogen, which also exert greater inhibitory effects on human GDH2 than on human GDH1. Oestrogens produced by astrocytes may play a role in controlling glutamate metabolism in the brain through their efficient interaction with human GDH2 (Borompokas et al., 2010). One of the variables affecting how GDH levels change as people age is sex. GDH activity was assessed in leukocytes from males and females from various age groups (Kravos and Malesic, 2010). The results revealed that leukocyte GDH activity decreased with age in both sexes, especially after the age of 50 years. Furthermore, after the age of 60 years, both sexes experienced accelerated decreases in GDH activity. GDH activity declined more rapidly in women between the ages of 20 and 50 than during menopause and showed a more uniform decline in men with aging. According to the results from that study, ageing may be exacerbated by a slow decrease in leukocyte GDH activity throughout life, and the brain appears to experience similar phenomena. Additional research on the importance of the effects of hormones on GDH activity and differences in GDH activity patterns between sexes is needed.

Finally, research has shown that an uncommon GLUD2 gene variant, T1492G, which causes a gain-of-function Ser445Ala substitution in hGDH2, is linked to the onset of early-onset Parkinson’s disease in some populations (Plaitakis et al., 2013). This variant has a significantly greater sensitivity to oestrogen-induced inhibition but is even more resistant to GTP inhibition than wild-type hGDH2. In female patients, the consequent regulation of hyperactive GDH by oestrogens may protect against the early onset of PD (Plaitakis et al., 2013). The positive results of oestrogen therapy in animal PD models provide more evidence supporting this hypothesis (Gillies and McArthur, 2010). In addition to a marked loss of neurons and reductions in the numbers of dendritic spines and axon terminals, transgenic mice overexpressing GDH also presented elevated levels and release of glutamate in the brain (Bao et al., 2009). Additionally, increased GDH expression led to the upregulation of numerous genes, including those linked to cellular damage, inflammation, oxidative stress, Parkinson’s disease, and Huntington’s disease (Wang et al., 2010). Early AD was reported to lead to a substantial increase in the quantity of 3-nitrotyrosine residues in proteins, including nitrosylated GDH, whose function is significantly diminished in some patients with early AD (Reed et al., 2009). However, additional research revealed that the GDH protein level in the brain is elevated (Burbaeva et al., 2005) and that the plasma of AD patients exhibits increased levels of GDH activity (Miulli et al., 1993).

## Conclusion

GDH is an essential metabolic enzyme that links glutamate to numerous other metabolic pathways. The roles of GDH dysregulation and dysfunction in a variety of illnesses, particularly neurodegenerative diseases, have received increasing attention in recent years. More investigations are needed to fully understand the effects of this enzyme, especially with respect to the neuroplasticity of the ageing human brain. We have examined the functions of GDH in the ageing brain in this review. We believe that GDH plays a key role in neuronal degenerative pathways. Future research efforts should consider human studies, even if the significance of GDH in ageing has been described. Promoting neuronal regeneration may help prevent neurodegenerative diseases. Future studies may uncover novel approaches for using GDH in their detection and treatment. Thus, developing new techniques to cure mental and neurological illnesses, slow the ageing process, and promote neuroplasticity may be possible.
